# Discovery and application of food catalysts to promote the coupling of PQQ (quinone) with amines

**DOI:** 10.3389/fnut.2024.1391681

**Published:** 2024-06-06

**Authors:** Kazuto Ikemoto, Satoko Imaruoka, Nur Syafiqah Mohamad Ishak

**Affiliations:** Niigata Research Laboratory, Mitsubishi Gas Chemical Company, Inc., Niigata, Japan

**Keywords:** PQQ, amino acid, food catalyst, HPLC, coupling, non-enzymatic, protein

## Abstract

**Background:**

Biocatalysts (enzymes) play a crucial role in catalyzing specific reactions across various industries, often offering environmentally friendly and sustainable alternatives to chemical catalysts. However, their catalytic activities are susceptible to denaturation. In this study, we present the discovery of novel protein-based biocatalysts derived from processed foods, including skimmed milk, soy milk, cheese, and dried tofu. These food catalysts exhibit high availability, low cost, safety, and thermo-stability.

**Results:**

Focusing on the physiologically intriguing coenzyme pyrroloquinoline quinone (PQQ), we observed that the reaction with glycine to form imidazolopyrroquinoline (IPQ) did not proceed efficiently when PQQ was present at very low concentrations. Surprisingly, in the presence of protein-based foods, this reaction was significantly accelerated. Notably, skimmed milk enhanced the PQQ detection limit (600 times lower) during high-performance liquid chromatography (HPLC) following IPQ derivatization. Milk appears to facilitate the reaction between PQQ and various amino acids, primary amines, and secondary amines. Further investigations revealed that food catalysis operates through a non-enzymatic mechanism. Additionally, nuclear magnetic resonance spectroscopy demonstrated that milk components interacted with amino substrates due to the ability of amines to react with quinones on colloidal surfaces.

**Conclusion:**

These practical food catalysts not only contribute to environmental safety but also hold significance across diverse scientific domains. Non-enzymatic protein catalysts find applications in biocatalysis, organic synthesis, food technology, analytical chemistry, and fundamental nutritional and evolutionary studies.

## 1 Introduction

Biocatalysis is a rapidly evolving research field. As a result, biocatalysts such as enzymes can now be efficiently designed using genetic engineering and applied to a variety of highly selective reactions in aqueous solutions (1, 2). The use of enzymes for high-performance liquid chromatography (HPLC) derivatization reactions has also been reported (3). However, enzymes suffer from two main drawbacks, namely, their high cost and the facile denaturation of their protein structures by heat, acids, bases, or other factors, which ultimately renders them inactive (4, 5).

Proteins are an important source of nutrients, and many foods contain proteins as a main component. Many of these foods are processed by heating, freezing, drying, salting, fermentation, seasoning, or the addition of additives. Importantly, these processes can also lead to enzyme deactivation. However, processed foods have a number of benefits, including increased shelf lives, reduced cooking times, and improved nutritional value, taste, and texture. Even though processed foods are in-expensive compared to purified enzymes and reagents, it is generally impossible to accelerate organic reactions in processed food media. The use of processed foods to promote reactions would therefore represent a significant breakthrough in the field of biocatalysis.

**Figure 1:**
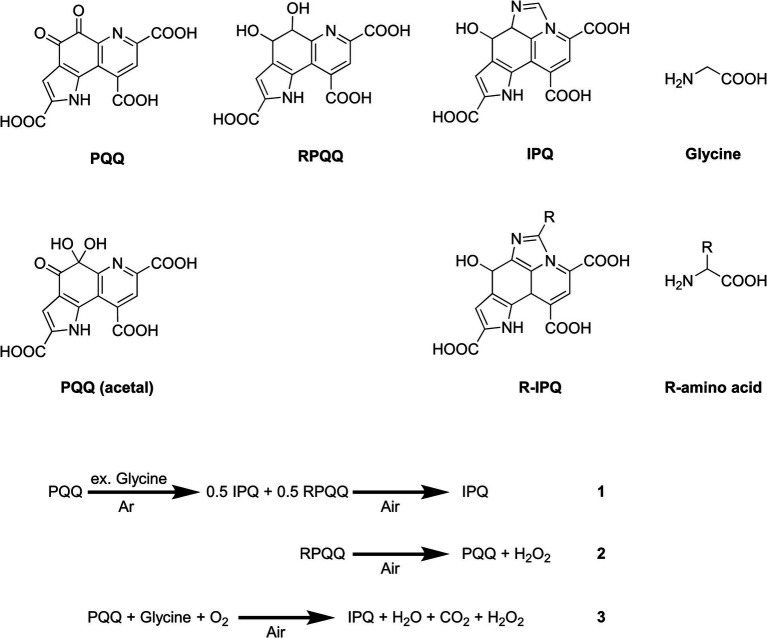
Chemical structures of the compounds of interest and the reaction process discussed herein.

Pyrroloquinoline quinone (PQQ) is a dehydrogenase coenzyme whose structure contains quinone, pyridine, pyrrole, and tricarboxylic acid moieties (Figure 1). This enzyme binds to calcium, magnesium, and lanthanoids to perform redox reactions (6). In addition, its quinone structure acts as the active site in the reduction of PQQ in the presence of a reducing reagent (7). In terms of its derivatives, the quinone and acetal forms of PQQ exist in equilibrium in an aqueous environment. PQQ molecules can undergo rapid redox cycles using glycine as a reducing agent under basic conditions, demonstrating a stronger redox potential than other redox molecules (8, 9). This reaction is utilized to estimate the concentration of PQQ in milk (9). It also has the function of protecting nerves from oxidative stress (10). In addition, PQQ disodium trihydrate, which is produced by fermentation (11, 12), is known to promote longevity (13), improve brain function (14) and suppress fat accumulation (15). To date, a range of analytical techniques have been employed for the detection of PQQ, including GC–MS (16, 17), HPLC–ultraviolet (UV) detection (18), HPLC–mass spectrometry (MS) (19), enzyme assays (20) and redox-based HPLC approaches (21). Despite its high stability in air and water, PQQ easily reacts with organic materials (such as under extraction conditions), which renders its quantitative analysis particularly challenging.

To address the above issues, our group previously developed a strategy to convert and analyze stable PQQ derivatives. More specifically, PQQ was reacted with amino acids to form imidazolopyrroquinoline (IPQ) (22) or imidazolopyrroquinoline with amino acid residues (R-IPQ) bearing an amino acid substituent (23) An analytical quantification method was developed based on the reaction between PQQ and glycine to generate the less reactive IPQ (24). The developed method was designed to proceed smoothly under neutral to alkaline pH conditions (25). However, when the concentration of PQQ was low, the reaction was hindered, thereby complicating the analysis. To date, no catalyst has been reported that promotes this reaction. The PQQ content has been measured in foods (17), tissues (16), milk (8), whey (8), and human milk (19). However, there have been no reports on the generation of IPQ from PQQ in this environment. This is because there are very few analyses that focus on measuring IPQ.

We have discovered a novel class of biocatalyst-food catalysts. This discovery joins the ranks of enzymes and ribozymes, and expands the field of biocatalysis. Food catalysts offer significant advantages over existing biocatalysts: (1) High affordability: food catalysts are significantly cheaper than traditional biocatalysts. (2) Easy availability: food sources are readily available, making them convenient for use. (3) Exceptional heat stability: food catalysts exhibit remarkable thermal stability compared with other biocatalysts. Furthermore, the study of non-enzymatic biological components as catalysts can deepen our understanding of material transformation in living systems. Notably, food catalysts are inherently safe and pose no environmental or health risks.

This report details the discovery of food catalysts that promote the reaction between PQQ and amino acids through a screening process. We also discuss the properties of these food catalysts and their underlying reaction mechanisms. Finally, this report demonstrates their application in HPLC analysis by derivatizing PQQ and enhancing the analytical sensitivity.

## 2 Materials and methods

### 2.1 Devices

HPLC analysis were used by Shimadzu Nexera XR or Shimadzu 10 AD. A JEOL 500 MHz NMR JNM-ECA500 spectrometer was used for all 1H NMR experiments. Particle size was used by HORIBA scientific nanoPartica SZ-100.

### 2.2 Reagents

All reagents were sourced from FUJIFILM Wako Pure Chemical Corporation, Japan, unless otherwise specified. As a pyrroloquinoline quinone (PQQ) disodium compound, BioPQQ^TM^ (Mitsubishi Gas Chemical Co., Inc.) was used. Imidazolopyrroquinoline (IPQ) and reduced PQQ (RPQQ) were synthesized according to previous reports (7, 23). All foods were general products (company and product name in supplementary materials).

### 2.3 HPLC analysis based on IPQ derivatization reaction

A carbonate buffer (50 mM) was prepared by diluting 250 mM carbonate buffer (15.95 g Na_2_CO_3_, 8.4 g NaHCO_3_, 1 L water) 5-fold with water. After mixing the desired sample specimen with a 10% glycine solution (20 mL) in a 50 mL tube under air, the solution was vigorously shaken at room temperature (20–25°C) for 1 h. Subsequently, an aliquot (0.2 mL) of this sample was mixed with 50 mM carbonate buffer (2 mL) in a 15 mL tube and shaken for 0.5 h. The sample was then analyzed using HPLC, according to the conditions outlined below. To investigate the reaction rate, only the first step was performed (i.e., shaking at room temperature for 1 h).

### 2.4 Catalyst screening test (foods list) (*n* = 3)

Sodium phosphate buffer (50 mM, pH 7) was used to prepare all solutions. Using a 2 mL reaction tube, the total reaction volume was 1 mL. The concentrations of reagents were as follows: 50 mg/L PQQ, 250 mg/L glycine, and 1 g/L catalyst. The reaction tube was heated for 2 h at 37°C. HPLC system equipped with a UV259 nm detector was employed. The basic conditions are the same as section 2.3. The changes are as follows. The eluent consisted of 50% methanol and 0.4% phosphoric acid. The column was an InertSustain C18 5 μm column (4.6 × 150 mm).

### 2.5 HPLC analysis (condition and system stability)

Conditions: A Shimadzu 10 AD HPLC system equipped with a UV259 nm detector was used. The eluent consisted of 100 mM acetic acid and 100 mM ammonium acetate (30:70, v/v, pH 5.1). The column was a YMC-Pack ODS-A column (0.99 × ⌀4.6 mm, 5 μm) operated at 40°C, and analysis was carried out over 30 min with an injection volume of 10 μL at a flow rate of 1.5 mL/min.

System stability: PQQ was used as a standard. The precision of the injection was determined by analyzing the standard material five times and passing the test if the area variation was less than RSD 0.5%. In the actual measurements, the RSD was 0.27%. The repeat precision was used to analyze nine different formulations of standard materials and passed the test with an RSD of less than 1.0%. The actual measurement showed an RSD of 0.56%. Quantitative analyses were performed using the USP method (26).

## 3 Results

### 3.1 Conversion of PQQ into IPQ

The reaction of PQQ with glycine forms IPQ, wherein the amino group reacts with the C=O bond of the quinone moiety to promote cyclodecarboxylation. IPQ is less reactive and more stable than PQQ, even in the presence of excess amino acids. Thus, the reaction of PQQ with excess glycine in an inert atmosphere was monitored using NMR spectroscopy (Supplementary Figure S1). It was found that IPQ and reduced PQQ (RPQQ) were produced. The subsequent bubbling of air through this reaction solution yielded only IPQ (Figure 1, Equation 1). This was accounted for by considering that RPQQ was reoxygenated to form PQQ (Figure 1, Equation 2) (25). With these considerations in mind, Equation 3 was derived, indicating that the complete conversion of PQQ to IPQ requires oxygen.

### 3.2 Screening of the food catalysts

**Figure 2:**
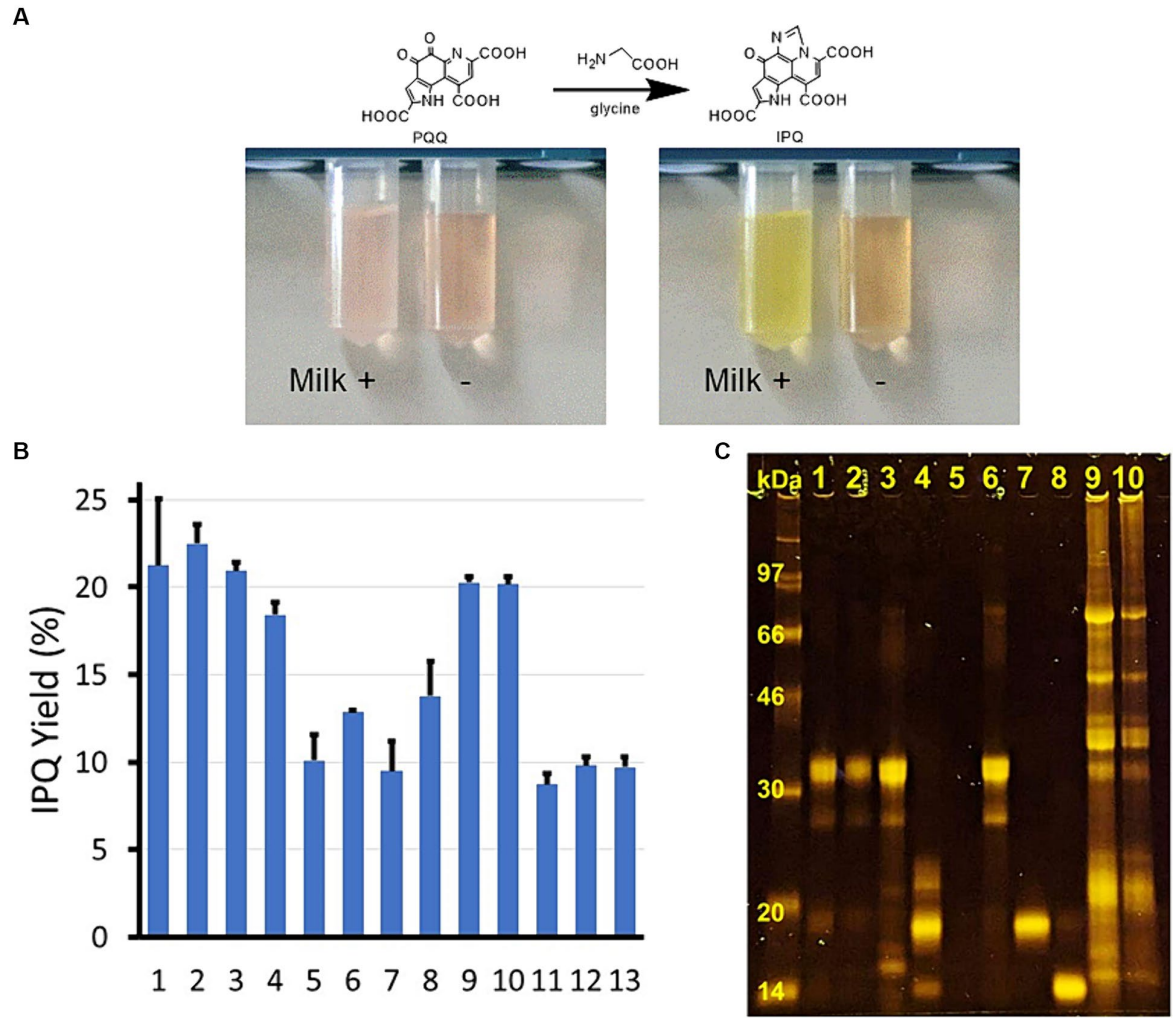
Food catalyst discovery, screening, and SDS–PAGE results. **(A)** Discovery of the food catalysts: left, after 0 h, and right, after 0.5 h at 37°C. Reaction conditions: PQQ, 117 μM; glycine, 0.133 M; ± whole milk powder, 1 g L^−1^. **(B)** IPQ yield (±SD) using different food samples as catalysts. 1 = whole milk powder, 2 = skimmed milk, 3 = cheese, 4 = whey protein, 5 = butter, 6 = casein, 7 = lactoglobulin, 8 = lactalbumin, 9 = soy milk, 10 = tofu (dried), 11 = starch, 12 = silica gel, and 13 = control (buffer). Reaction conditions: 37°C, 2 h; food sample, 1.0 g L^−1^ (for soy milk, the concentration was adjusted based on the solids content); PQQ, 117 μM; glycine, 3.3 mM; sodium phosphate buffer, 50 mM, pH 7.0. **(C)** Protein analysis (SDS–PAGE) of different food samples.

During the development of an analytical approach for PQQ derivatization based on the above reaction, we found PQQ in some foods was converted to IPQ more easily than that in a pure form. In particular, the reaction was accelerated in the presence of whole milk powder (Figure 2A). Thus, the components responsible for promoting this reaction were initially investigated. The amino acid concentration was kept low during the reaction to observe the effects of the additives more easily. As outlined in Figure 2B and Supplementary Table S1, starch was also investigated as a carbohydrate, lactoglobulin and lactalbumin were examined as pure proteins, and soymilk and tofu (dried) powder were employed as soybean-derived foods. Various food products promoted the PQQ derivatization reaction, with significantly higher yields (2×) being obtained in the presence of whole milk powder, skimmed milk, cheese, whey protein, soymilk, and tofu (dried) powder. Improved yields were also obtained using casein and lactalbumin (1.4–1.5×), whereas no improvements were observed using butter, starch, or lactoglobulin.

### 3.3 Protein analysis (SDS–PAGE)

Since protein-rich foods were found to effectively pro-mote the reaction, all food specimens of interest were subjected to sodium dodecylsulfate polyacrylamide gel electrophoresis (SDS–PAGE) for protein analysis (Figure 2C). The whole milk powder contained casein and lactoglobulin, the skimmed milk and cheese were primarily composed of casein, and the whey protein mainly com-prised lactoglobulin with a minor amount of lactalbumin. Butter contained very few proteins, while the soymilk and tofu (dried) specimens comprised mixed proteins with various molecular weights.

### 3.4 Effect of reaction conditions

After heating the skimmed milk and tofu (dried) pow-der specimens in the buffer solution at 80°C for 30 min, comparable IPQ yields were obtained. The reaction was also repeated under an Ar atmosphere, giving IPQ yields of 22.5% (skimmed milk) and 20.2% (tofu, dried), which were significantly higher than the 9.3% yield obtained for the control (buffer). Comparable yields were obtained under both sets of conditions (i.e., air and Ar gas). Thus, as a model experiment, a primary amine (aminoethanol), 81.9 mM (5 g L^−1^) and secondary amine (N-methylamino ethanol), 66.6 mM (5 g L^−1^) were reacted with PQQ (2.34 mM) in the presence of skimmed milk (2 g L^−1^) under an Ar atmosphere at 37°C. In the case of ethanolamine, the PQQ conversion reached 61.0% after 1 h for the reaction containing skimmed milk (c.f., 44.5% for the control), while a conversion of 43.8% (21.9% control) was obtained for the N-methylamino ethanol reaction after 3 h. These results clearly indicate skimmed milk accelerated the addition of amines to the quinone carbonyl moiety.

### 3.5 Investigation of the amino acid scope

As previously reported, substituted IPQ can be obtained by reacting various amino acids with PQQ (17). Thus, the corresponding reactions with leucine, isoleucine, valine, asparagine, and arginine were investigated, both in the presence and absence of skimmed milk (60°C for 3 h). The HPLC results are summarized in Supplementary Figure S2 and Table 1.

**Table 1:** Reaction of various amino acids with PQQ at 60°C for 3 h*.

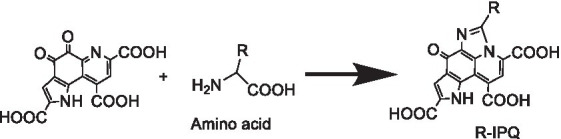			
Amino acid	R-IPQ yield (milk)	R-IPQ yield (control)	Ratio (milk +/−)
Leu	54.0	21.3	2.5
Ile	86.5	37.1	2.3
Val	59.3	25.5	2.3
Asn	13.2	4.8	2.7
Arg	36.7	12.3	3.0

*Reaction conditions (2 mL reaction tube, 1 mL total volume): PQQ, 2.34 mM; amino acid, 5 g L^−1^ (Leu 38.1, Ile 38.1, Val 42.7, Asn 37.8, Arg 28.7 mM); and skimmed milk, 2 g L^−1^ in water. The arginine solution was adjusted to pH 5 using phosphoric acid.

### 3.6 HPLC analysis of PQQ derivatization reaction

**Figure 3:**
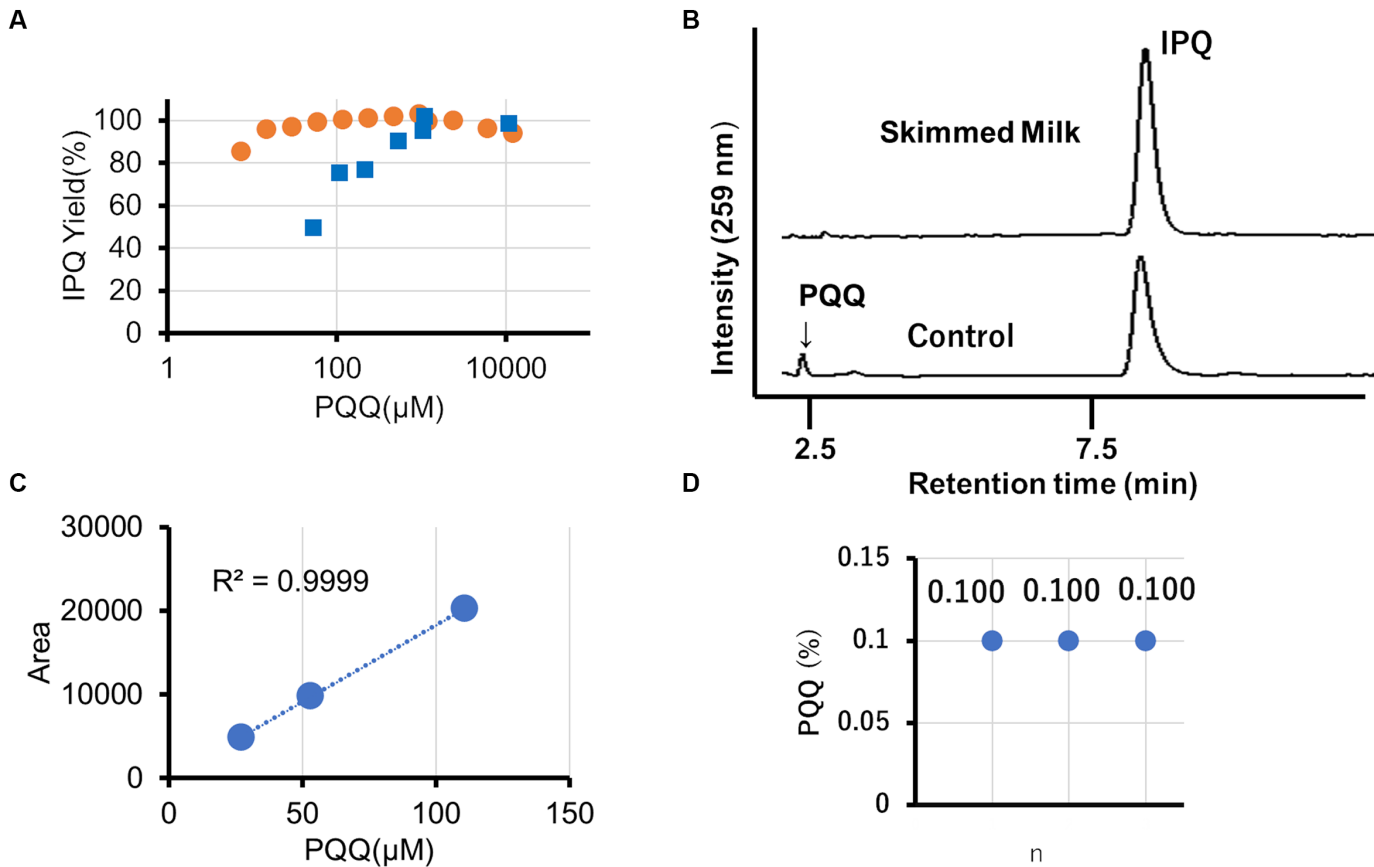
HPLC analysis of the PQQ derivatization reaction carried out in the presence of skimmed milk. **(A)** Effect of the PQQ concentration on the IPQ yield in 1.33 M (10 wt%) glycine (blue: no catalyst; orange: 1 wt% skimmed milk catalyst). **(B)** Chromatographs for reaction solutions with and without skimmed milk using an initial PQQ concentration of 117 μM. **(C)** Influence of PQQ concentration on the HPLC peak area in the presence of skimmed milk. **(D)** Validation of PQQ analysis (*n* = 3) in chocolate.

The derivatization of PQQ using glycine to form stable IPQ facilitates the analysis of reactive PQQ (Supplementary Figure S3). A two-step reaction was conducted (pH 6: PQQ → IPQ + RPQQ; pH 10: reoxygenation of RPQQ) (Supplementary Figure S4). Notably, large excesses of glycine and catalyst were used to ensure that the reaction reached completion. In the abssence of a catalyst, PQQ concentrations of 10,000, 500, and 50 μM gave IPQ yields of 95, 90, and 50%, respectively, whereas in the presence of 1 wt % skimmed milk as the catalyst, IPQ yields of 99 and 96% were obtained at PQQ concentrations of 50 and 15 μM PQQ, respectively (Figure 3A). These results demonstrate the significant catalytic action of skimmed milk proteins. Chromatographic analysis confirmed that IPQ was the main product, with no other interfering peaks observed (Figure 3B). This suggests that this method is suitable for the quantitative analysis of PQQ. We performed the analysis with reference to the standards for quantitative analysis methods of the United States Pharmacopeia (USP) (26). The precision of this analysis was less than 1% relative standard deviation. Three chocolate samples, each containing 0.1 wt % PQQ (PQQ in extract solution: 58 μM), were analyzed using this approach. Importantly, the calibration curve constructed for the developed method was linear in the concentration range of 25–110 μM with an R2 value of 0.999 (Figure 3C). All three analyzed samples were found to contain 0.1 wt % PQQ (Figure 3D; Supplementary Table S2), which corresponded to 100% recovery. No variations were observed in analytical values.

### 3.7 Investigation of reaction mechanism

**Figure 4:**
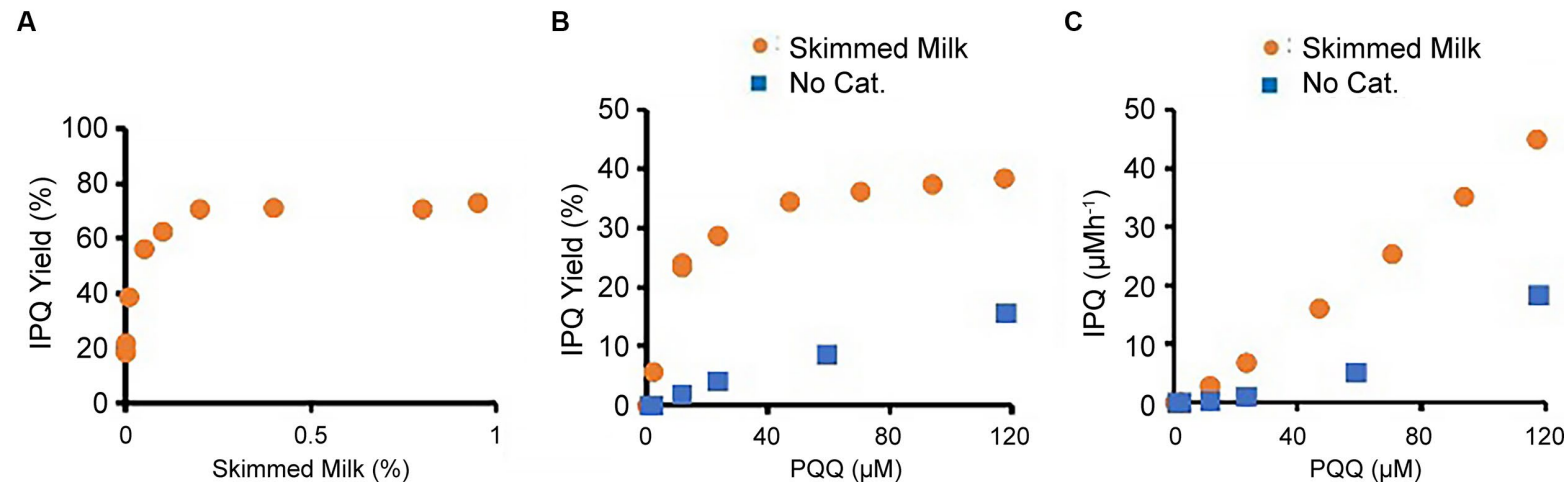
Effects of PQQ and skimmed milk concentrations on derivatization reaction. **(A)** Effect of skimmed milk concentration (PQQ, 117 μM; glycine 0.133 M). **(B)** Effect of PQQ concentration. **(C)** Relationship between PQQ and IPQ concentrations. Note that **(B)** and **(C)** use the same data but with different vertical axes (IPQ yield (%) and IPQ concentration (μM/h), respectively).

The effects of different reactant/reagent concentrations on the reaction were subsequently studied. For this purpose, the glycine concentration was reduced to 0.133 M (10 g L − 1), and the effects of milk supplementation were examined over a reaction time of 1 h (Figure 4A). In the presence of 117 μM PQQ, the IPQ yield was 19% in the absence of a catalyst. However, the addition of 0.01 wt % skimmed milk doubled the yield to 39%, while the addition of 0.2 wt % skimmed milk gave a yield of 71%. Increasing the skimmed milk loading further had little effect on the IPQ yield. Next, the effect of the initial concentration of PQQ was studied using a 0.133 M aqueous glycine solution, 0.01 wt % skimmed milk, and reaction time of 1 h (Figures 4B,C). In the absence of a catalyst, the relationship between the PQQ concentration and IPQ yield was almost linear. In contrast, in the presence of skimmed milk, the IPQ yield increased rapidly as the PQQ concentration increased and then plateaued at higher PQQ concentrations. This corresponds to a linear in-crease in IPQ concentration against initial PQQ concentration, as plotted in Figure 4C.

### 3.8 1H NMR analysis

**Figure 5:**
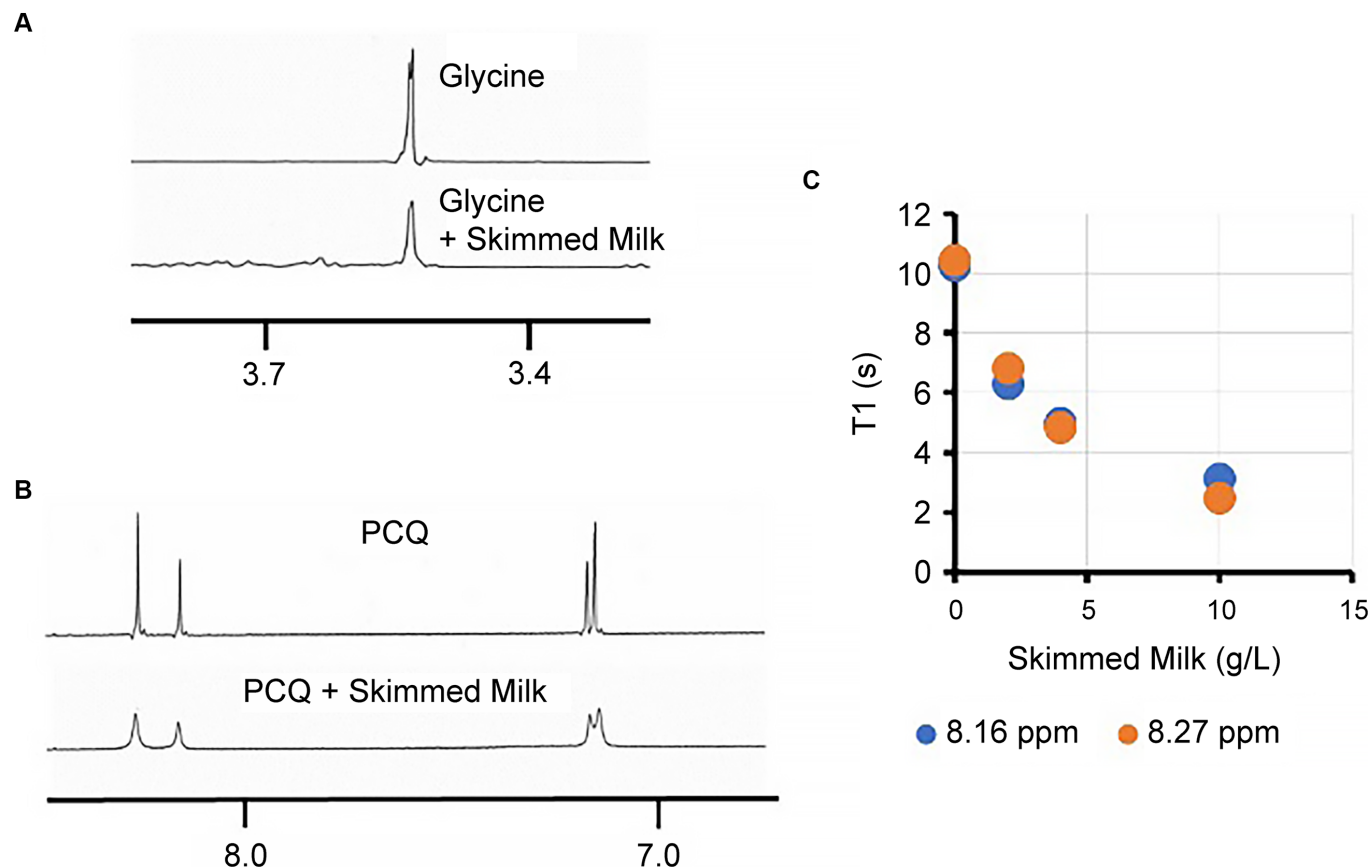
Effects of skimmed milk addition on the glycine and PQQ ^1^H NMR spectra. **(A)** ^1^H NMR peak of glycine in the presence and absence of skimmed milk. **(B)** ^1^H NMR peaks of PQQ in the presence and absence of skimmed milk. **(C)** *T*_1_ relaxation times for the aromatic PQQ peaks.

The intermolecular interactions between large skimmed milk molecules and small molecules can be observed using NMR based on peak broadening and changes to the relaxation time. Skimmed milk is a colloidal solution with a particle size of approximately 1,200 nm, while the particle size of casein is 520 nm (Supplementary Figure S5). Upon the addition of skimmed milk to the glycine solution, a broadening of the NMR peaks was observed (Figure 5A). Since PQQ exists in an equilibrium between its PQQ (acetal) and quinone forms in water, four peaks were observed in the aromatic region when the solution was prepared in D2O. Notably, the peaks corresponding to PQQ broadened upon the addition of skimmed milk (Figure 5B). Relaxation time measurements were performed for the two low-field aromatic peaks of PQQ, which corresponded to the pyridine ring protons of the quinone and acetal forms. The T1 re-laxation times of these peaks significantly reduced in the presence of skimmed milk (Figure 5C), demonstrating that both PQQ species interacted with skimmed milk.

## 4 Discussion

High-protein foods, such as whole milk powder, skimmed milk, cheese, and whey protein, were the most effective catalysts for the derivatization reaction. Soymilk and tofu (dried) exhibited slight reaction-promoting effects, while butter, starch, and silica gel (an inorganic substance) had no effect on the reaction. Based on the compositions of these foods, it was considered that proteins were responsible for catalyzing the derivatization reaction. The main protein identified in the whole milk powder (essentially milk with the water removed), skimmed milk (whole milk with the fat removed) (27) and cheese (denatured and coagulated milk proteins) sample was casein, whereas that in the whey protein sample (an isolated product with high protein purity) was lactoglobulin, which cannot be coagulated. In contrast, the butter specimen contained very little protein (Supplementary Table S1). With these considerations in mind, it was clear that high-protein foods were the most active in promoting the reaction. However, when the reaction was repeated using purified casein and lactalbumin, the conversions were negligible, and no response enhancement was observed upon treatment with lactoglobulin. Interestingly, soymilk and tofu (dried) both accelerated the reaction. These foods contained mixtures of proteins, including some denatured ones. They are prepared by adding water to soybeans and boiling (soymilk) or by coagulating soymilk using Ca and Mg, repeatedly freezing and thawing, and removing the water (tofu, dried) (28). Proteins that undergo coagulation due to heating, freezing, and thawing typically lose their original tertiary structures, so the fact that these foods exerted a catalytic effect is particularly interesting.

**Figure 6:**
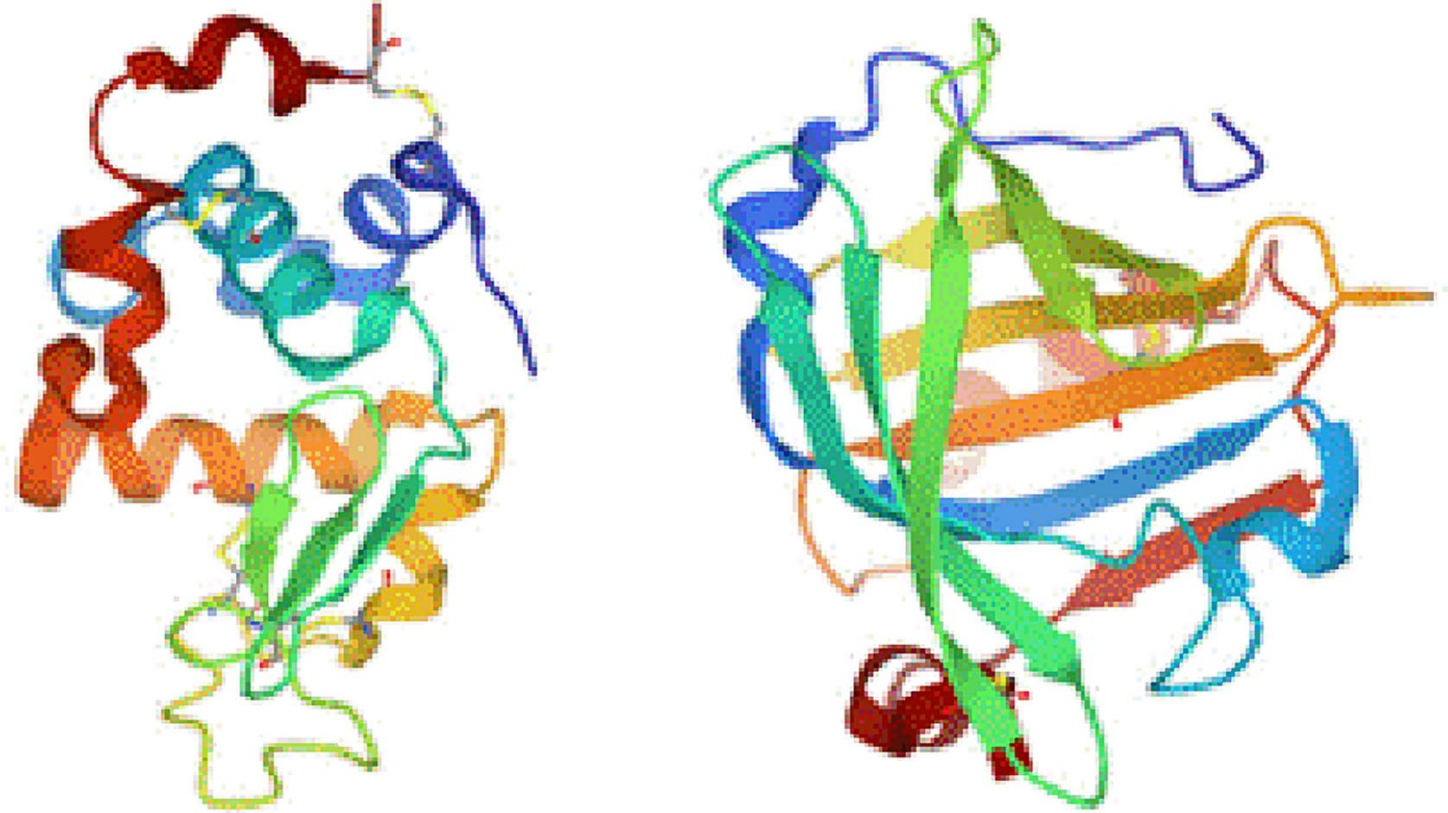
Three-dimensional structures of pure proteins: (left) lactalbumin and (right) lactoglobulin.

Subsequently, the structure–activity relationships were examined for the pure proteins. First, the structures of lactalbumin and lactoglobulin were compared, considering that lactalbumin was more active than lactoglobulin. The structures were modeled using data from the Protein Data Bank and the HOMCOS molecular modeling server. The results showed that the molecules in contact with lactoglobulin were fat-soluble compounds such as retinol, retinoic acid, and octanoic acid. Furthermore, lactalbumin forms an α-helical structure, whereas lactoglobulin forms a β-sheet structure (Figure 6).

Because α-helices are more hydrophilic than β-sheets, these structural differences may be responsible for the observed differences in activity. However, although the active foods are hydrophilic, and the reactive surfaces of their proteins appear hydrophilic, these considerations are insufficient to explain the factors contributing to the detected high activities. Docking simulation was performed using Autodock Vina (Supplementary Table S3) to investigate the affinity between small molecules and proteins. The result of docking scores indicates that the binding affinities of PQQ and IPQ were more stable with lactalbumin compared to with lactoglobulin. For glycine, the affinity of the two proteins is small. Therefore, adsorption of PQQ easily progresses with lactalbumin rather than lactoglobulin, thereby cause difference in catalytic activity.

It was surprising that while whole milk powder, skimmed milk, cheese, and whey protein demonstrated high activities, purified casein, lactalbumin, and lactoglobulin did not. This result is of particular importance since it implies that these foods could be applied as active catalysts with fewer purification steps. The types of proteins and protein contents of the active foods varied from 23% for whole milk powder to 97% for whey powder. This indicates that the activity was not caused by a specific protein content or structure. Moreover, the difference in colloidal size between the food and protein molecules in water (e.g., skimmed milk: 1200 nm; casein: 520 nm) indicates that the surface properties are likely more important than the surface area in determining the activity. It was therefore assumed that food molecules can be-come colloidal during mixing or denaturation, and that the catalytic reactions occur on the surfaces of the colloidal particles.

Because the investigated food specimens contain other components that possess a range of functional groups, it is possible that these compounds played a role in deter-mining the catalytic activity. This could be comparable to the effects of inorganic compounds with abundant active surface sites. Such an assumption could be supported by the fact that the catalytic activity remained stable at temperatures up to 80°C, which is a significant advantage in terms of potential applications.

To demonstrate the potential applicability of these food-based catalysts, other amino acid reactions were investigated. The reaction was promoted when using these amino acids, including extremely bulky branched-chain amino acids and alkyl side chain-free amino acids such as arginine and asparagine. Importantly, the product yields were more than twice those recorded in the absence of any catalyst. Despite attempts to induce an enzyme-like reaction using skimmed milk, a linear relationship was observed between the PQQ concentration and IPQ molar yield, indicating the absence of Michaelis—Menten kinetics (29). These reactions are therefore non-enzymatic. Indeed, the catalytic activity of the skimmed milk system was particularly low compared to those of well-known enzymes. However, skimmed milk is significantly cheaper; therefore, it is still promising to replace enzymes in some situations. In fact, skim milk costs $0.11/g (amazon) compared to $49 (Wako) for the representative enzyme trypsin. Enzyme technology has made remarkable progress, enabling high-precision and environmentally friendly manufacturing processes in various fields (30). There have been no reports of organic synthesis using food as a catalyst, and organic synthesis using food additives as catalysts has been studied (31). Because food is cheaper and more sustainable than food additives, utilizing food as a catalyst could lead to the development of greener synthetic methods.

The reaction between PQQ and IPQ involves several steps. To investigate the reaction in greater detail, a primary amine (ethanolamine) and secondary amine (methylethanolamine) were employed as model compounds for the reaction in an inert atmosphere. The PQQ conversion rates increased for both the primary and secondary amines in the presence of skimmed milk, further confirming that food-based catalysts promote the addition of amino groups to the quinone carbonyl groups of PQQ.

The NMR peaks of PQQ (in its equilibrium state) broadened in the presence of amino acids, indicating that inter-actions occur between skimmed milk, PQQ, and amino acids. The T1 relaxation time is inversely proportional to molecular motion, wherein a slower molecular motion corresponds to a shorter T1 relaxation time (32) Skimmed milk therefore appears to reduce the relaxation times of both the quinone- and acetal-type PQQ structures, likely because the movement of both chemical species is sup-pressed by the skimmed milk colloid. This result supports the formation of a PQQ–skimmed milk complex.

**Figure 7:**
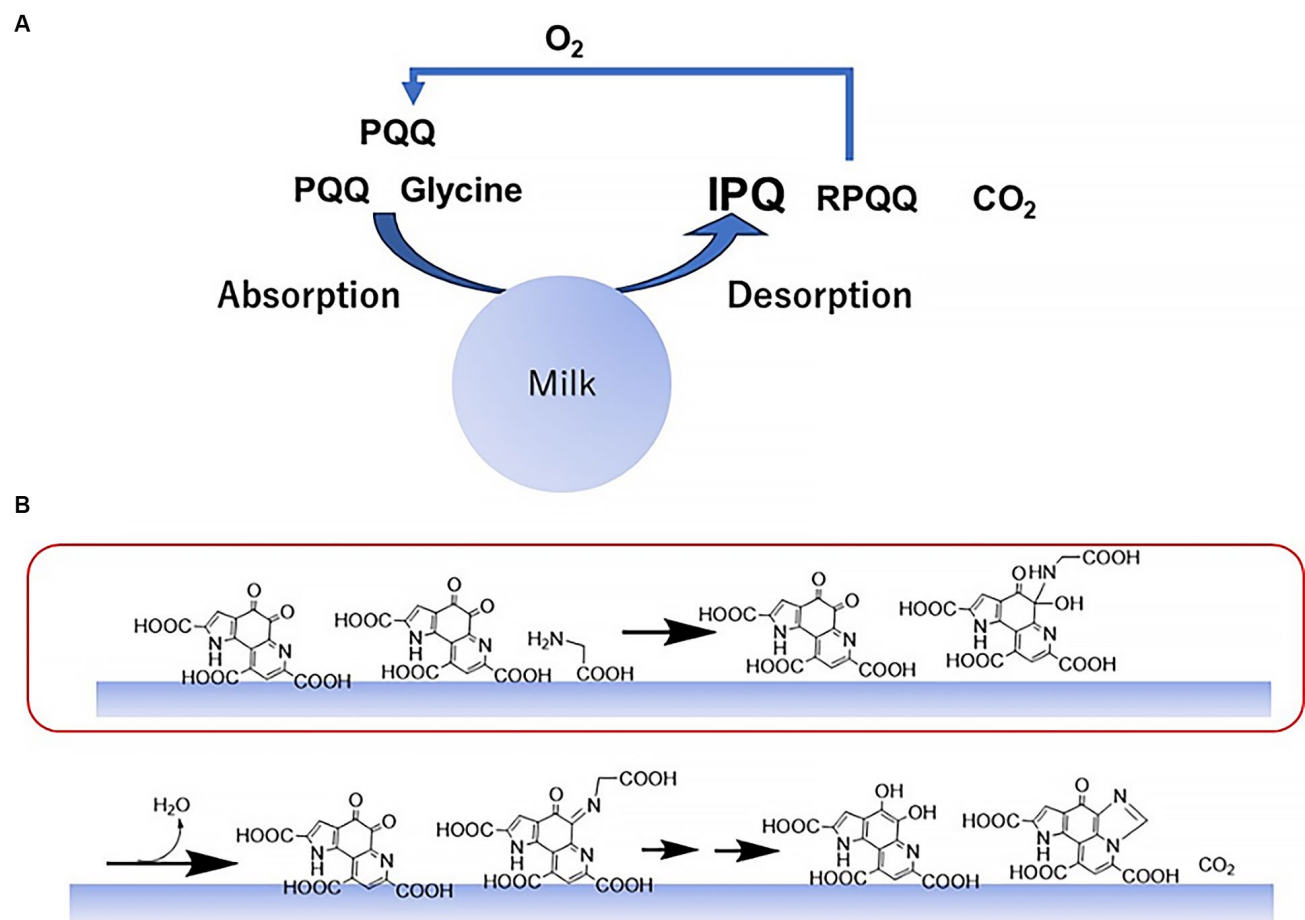
Mechanism for the reaction between PQQ and glycine promoted by skimmed milk. **(A)** Overall reaction mechanism. **(B)** Reaction steps on the surfaces of the colloidal milk particles. Amine addition to the carbonyl group is accelerated by the food catalyst (red box).

To summarize these results, a plausible reaction mechanism is illustrated in Figure 7. Initially, glycine and PQQ are adsorbed onto the surface of the colloidal skimmed milk particles and react on the milk surface. This results in the generation of IPQ, RPQQ, and CO_2_, wherein the produced RPQQ is reoxygenated to PQQ in the presence of oxygen. Subsequently, on the surfaces of the milk particles, the amine binds to the PQQ quinone moiety; this is the main reaction step promoted by the food catalyst. Intramolecular dehydration then proceeds, and subsequent Schiff base formation and cyclodecarboxylation generate IPQ and RPQQ.

Micelles are well-known colloids. Several studies have reported that micelles can act as catalysts (33). Recent research has focused on the utilization of hydrophobic reaction sites in aqueous environments (34). In addition, studies have shown that micelle interfaces can act as catalysts. The reaction substrates were concentrated on the surface of the micelles (35). We hypothesized that a similar phenomenon would occur with food catalysts. The increased concentration of substrates on the surfaces of food colloids may lead to catalysis. This hypothesis aligns with the NMR spectroscopy results and observation that the reaction proceeds at low concentrations. As a model for chemical evolution, the condensation products of amino acids can promote ATP hydrolysis (36). It has also been reported that amyloid self-replicates (37). Furthermore, cases in which antibodies act as catalysts have been reported (38). Many inorganic catalysts exhibit catalytic activity without a catalyst design. We believe that it is possible for food colloids to exhibit catalytic activity.

The quinone group of PQQ is extremely reactive to amino acids, which can lead to inaccurate HPLC analysis when determining the PQQ content. The analysis of capsules containing both PQQ and various amino acids is therefore difficult. Derivatization is therefore required, and in the case of the current study, the low-cost and non-toxic glycine was selected for reaction under mild conditions. The results demonstrate that the developed method, based on derivatization and the addition of a skimmed milk catalyst, is suitable for combination with HPLC analysis. Importantly, this method is also suitable for systems containing low PQQ concentrations, with a linear calibration curve being obtained, even under such conditions. The standard PQQ intake by a single person is approximately 20 mg/day in capsules (39). The addition of pyrroloquinoline quinone (PQQ) to large volumes of food, such as chocolate, can lead to a decrease in its concentration, making the analysis difficult. However, derivatization can increase the detection sensitivity by a factor of 600, making it a valuable tool for HPLC analysis and other highly sensitive methods (HPLC–MS, HPLC-MS–MS). Thus, the development of analytical approaches for the determination of trace levels of PQQ, such as that reported herein, is essential in the context of commercial applications.

While previous research on milk has focused on PQQ content and its enzymatic oxidation of whey sugars (40), IPQ analysis remains rare. Therefore, food catalysts have not been developed yet. Many foods promote non-enzymatic interactions between PQQ and various amino compounds. The rapid redox cycle under alkaline conditions and IPQ promotion are the same initial steps. We hypothesized that the body harbors an abundance of proteins analogous to those of food catalysts. These putative proteins could potentially facilitate an even faster PQQ redox cycle or promote the formation of the reduced form of PQQ, ultimately leading to an enhanced physiological activity. PQQ derivatives are produced in cells (41). Although PQQ is abundant in breast milk (42), its levels are likely to be lower than expected, because milk boosts the reactivity of PQQ. The phenomenon of reaction promotion by protein-based substances that are not enzymes is important to understand the natural distribution of PQQ and the mechanisms of its physiological activity. Food catalysts are highly available and can be deployed “from the kitchen to the laboratory.” The search for nonenzymatic catalysts from processed foods is a frontier in scientific research. Organic chemistry offers new organocatalysts and analytical chemistry offers derivatization methods that are safe for the environment and researchers. There is a possibility of understanding the metabolism of low-molecular-weight compounds, which has not yet been explained in the fields of medical pharmacy and biochemistry, and of synthesizing prodrugs using non-enzymatic catalysis. In the food industry, we have created new processing technologies. Advances your understanding of chemical evolution. This scientific result is expected to be safe, environmentally compatible, and cost competitive when industrialized.

## 5 Conclusion

Denatured foods, such as skimmed milk, can promote catalytic reactions in a non-enzymatic manner. Using this biocatalyst, the derivatization of PQQ using amino acids proceeded efficiently even at low PQQ concentrations. This method was deemed suitable for the derivatization of PQQ prior to analysis by HPLC. The results of this re-search represent a new advance in the area of biocatalysis, namely food catalysts, which are advantageous in terms of their stability, price, and availability.

## Data availability statement

The raw data supporting the conclusions of this article will be made available by the authors, without undue reservation.

## Author contributions

KI: Visualization, Resources, Project administration, Writing – review & editing, Writing – original draft, Investigation, Conceptualization. SI: Investigation, Writing – original draft. NI: Formal analysis, Writing – original draft.
