# Supplementary material for: Discovery and application of food catalysts to promote the coupling of PQQ (quinone) with amines
Source: Front Nutr. 2024 Jun 6;11:1391681. doi: 10.3389/fnut.2024.1391681 (PMC11187273; doi:10.3389/fnut.2024.1391681)
Supplement: Supplementary file 1 [file Data_Sheet_1.DOCX]

Original Research articles

Supplementary Material

Discovery and Application of Food Catalysts to Promote the Coupling of PQQ (Quinone) with Amines

Kazuto Ikemoto ^1^*, Satoko Imaruoka ^1^, Nur Syafiqah Mohamad Ishak ^1^

^1^ Niigata Research Laboratory, Mitsubishi Gas Chemical Company, Inc., 182, Tayuhama, Kita-ku, Niigata City, Niigata 950-3112, Japan

Supplementary Material

# Experimental section

## Reagents

All reagents were sourced from FUJIFILM Wako Pure Chemical Corporation, Japan, unless otherwise specified. As a pyrroloquinoline quinone (PQQ) disodium compound, BioPQQ^TM^ (Mitsubishi Gas Chemical Co., Inc.) was used. Imidazolopyrroquinoline (IPQ) and reduced PQQ (RPQQ) were synthesized according to previous reports.^1,2^ Deionized water was used for all experiments. All foods employed are listed below.

|  | **Food** | **Commercial source and product name** |
| --- | --- | --- |
| 1 | Whole milk powder | Yotsuba Milk Products Co., Ltd., Japan, Hokkaido Milk powder |
| 2 | Skimmed milk | FUJIFILM Wako Pure Chemical Corporation, Japan |
| 3 | Natural cheese | Megmilk Snow Brand Co., Ltd., Japan, Kraft 100% Parmesan cheese |
| 4 | Whey protein | NICHIGA, Japan, Grass fed WPI instant whey protein |
| 5 | Butter | Megmilk Snow Brand Co., Ltd., Japan, Yukijirushibutter (unsalted) |
| 6 | Casein | FUJIFILM Wako Pure Chemical Corporation, Japan |
| 7 | Lactoglobulin | Sigma-Aldrich, US |
| 8 | Lactalbumin | Sigma-Aldrich, US |
| 9 | Soy milk | Kikkoman, Japan, Processed soy milk |
| 10 | Dried tofu | Misuzu, Japan, Powdered Tofu (Tofu meal) |
| 11 | Starch | FUJIFILM Wako Pure Chemical Corporation, Japan |
| 12 | Silica gel | FUJIFILM Wako Pure Chemical Corporation, Japan |

## Reaction conditions

### Catalyst screening test (foods list)

Sodium phosphate buffer (50 mM, pH 7) was used to prepare all solutions. Using a 2 mL reaction tube, the total reaction volume was 1 mL. The concentrations of reagents were as follows: 50 mg/L PQQ, 250 mg/L glycine, and 1 g/L catalyst. The reaction tube was heated for 2 h at 37 °C using an aluminum block. Subsequently, the tube was cooled on ice and the reaction solution was analyzed by high-performance liquid chromatography (HPLC).

### Reaction of PQQ and various amino acids

Using a 2 mL reaction tube, the total reaction volume was 1 mL. The concentrations of reagents were as follows: 1 g/L PQQ, *X* g/L amino acids, and 2 g/L skimmed milk in water. The amino acids used were Leu, Ilu, Val, and Asn (*X* = 5 g/L) and Arg (*X* =2 g/L). The pH of the aqueous solution was adjusted to 5 using phosphoric acid. The reaction mixture was heated for 3 h at 60 °C using an aluminum block, after which the tube was cooled on ice and the reaction solution was analyzed by HPLC.

## HPLC analysis based on IPQ derivatization reaction

A carbonate buffer (50 mM) was prepared by diluting a 250 mM carbonate buffer (15.95 g Na_2_CO_3_, 8.4 g NaHCO_3_, 1 L water) 5-fold with water. After mixing the desired sample specimen with a 10% glycine solution (20 mL) in a 50 mL tube under air, the solution was vigorously shaken at room temperature (20–25 °C) for 1 h. Subsequently, an aliquot (0.2 mL) of this sample was mixed with the 50 mM carbonate buffer (2 mL) in a 15 mL tube and shaken for 0.5 h. The sample was then analyzed by HPLC according to the conditions outlined below. For investigation of the reaction rate, only the first step was carried out (i.e., to shaking at room temperature for 1 h).

### HPLC conditions

Condition 1

For catalyst screening, a Shimadzu Nexera XR HPLC system equipped with a UV259 nm detector was employed. The eluent consisted of 50% methanol and 0.4% phosphoric acid. The column was an InertSustain C18 5 µm column (4.6 × 150 mm) and analysis was carried out with an injection volume of 10 μL at a flow rate of 1.0 mL/min.

Condition 2

For sample analysis and mechanistic investigations, a Shimadzu 10AD HPLC system equipped with a UV259 nm detector was employed. The eluent consisted of 100 mM acetic acid and 100 mM ammonium acetate (30:70 v/v, pH 5.1). The column was a YMC-Pack ODS-A column (0.99 × ⌀4.6 mm, 5 μm) operated at 40 °C, and analysis was carried out over 30 min with an injection volume of 10 μL at a flow rate of 1.5 mL/min.

## SDS-PAGE

For SDS-PAGE experiments, the ATTO EzLabel FluoroNeo labeling kit (WSE-7010) was employed. Initially, a master mix was prepared by adding an aliquot (40 μL) of the sample buffer (5× conc.) in the kit to water (60 μL) and the labeling reagent (2 μL). Subsequently, an aliquot of the master mix (20 μL) was mixed with the desired sample solution (20 μL, 1 g/L in phosphate-buffered saline (PBS), 10 g/L for soy milk and tofu). The resulting mixture was heated at 95 °C for 3 min, after which a reducing agent (DTT, 1 μL) was added, with a further 3 min of heating at 95 °C. SDS-PAGE was carried out using an ATTO Compact PAGE Ace WSE 1010 instrument containing ATTO ePAGEL-HR (12.5%) in the electrophoresis tank. The running buffer was the Nippon Gene SDS-PAGE Running buffer. An aliquot (1 μL) of the sample solution was added, and the experiment was run for 30 min.

## ^1^H NMR measurements

A JEOL 500 MHz NMR JNM-ECA500 spectrometer was used for all ^1^H NMR experiments. For the interaction measurements, all spectra were recorded in D_2_O (Sigma-Aldrich) containing 100 mM PBS buffer at pH 7. Sodium trimethylsilyl propionic acid (TSP) was used as the external standard. The concentrations of compounds were as follows: glycine, 4 g/L; glycine, 4 g/L + skimmed milk, 10 g/L; PQQ, 4 g/L; and PQQ, 4 g/L + skimmed milk, 10 g/L. The *T*_1_ relaxation times were measured at various skimmed milk concentrations with 4 g/L PQQ. The results provide insight into the relationship between skimmed milk and PQQ.

### Conversion of PQQ to IPQ

PQQ (23 mg) was mixed with 10% glycine in D_2_O (0.6 mL) including TSP. The sample was centrifuged to remove any undissolved solids. The resulting mixture was stored in an NMR tube in a nitrogen atmosphere at room temperature for 2 d prior to measurement. The sample was measured by NMR in a nitrogen atmosphere. After the measurement, air flow (2.5 mL/min) was bubbled through the NMR solution overnight, after which the sample was remeasured.

## Analysis of chocolate

### Sample preparation

For the analysis of chocolate specimens, commercially available chocolate containing PQQ was melted at 50 °C. BioPQQ^TM^ was then added to give a concentration of 0.1%, and the resulting mixture was placed in a refrigerator for solidification. Subsequently, the solidified sample was crushed in a mortar as finely as possible. A sample of the crushed chocolate (0.5 g) was added to a 50 mL reaction tube along with a glycine/skimmed milk solution (10% and 1%, respectively, 20 mL total). After heating for 15 min at 50 °C, the mixture was homogenized by vortexing for 30 s. The mixture was then shaken at 200 rpm for 60 min at room temperature and subjected to centrifugation to remove all solids (3000 rpm, 10 min, 50 mL tube → 12,000 rpm, 5 min, 1 mL tube). An aliquot of the resulting sample (0.2 mL) was mixed with 250 mM carbonate buffer (pH 10, 2 mL) in a 15 mL tube and shaken at 160 rpm for 30 min. The solution was then diluted 5-fold using the HPLC eluent and the sample was filtered prior to analysis (HPLC condition 2).

## Protein modeling

The protein structures of lactalbumin and lactoglobulin were modeled using data from the International Protein Data Bank (PDB, https://www.rcsb.org/) and the HOMCOS server (HOMology modeling of COmplex Structure, https://homcos.pdbj.org/). The HOMCOS server was used to search for conformational data of homologous proteins bound to other molecules based on amino acid sequences.

Structural data of lactalbumin and lactoglobulin

| **Molecule** | **Chains** | **Sequence Length** | **PDB DOI** |
| --- | --- | --- | --- |
| α-lactalbumin | [A](https://www.rcsb.org/sequence/7WQG#A), [B](https://www.rcsb.org/sequence/7WQG#B), [C](https://www.rcsb.org/sequence/7WQG#C), [D](https://www.rcsb.org/sequence/7WQG#D), [E](https://www.rcsb.org/sequence/7WQG#E), [F](https://www.rcsb.org/sequence/7WQG#F) | 121 | [10.2210/pdb7WQG/pdb](http://doi.org/10.2210/pdb7WQG/pdb) |
| β-lactoglobulin | [A](https://www.rcsb.org/sequence/5LKE#A) | 162 | [10.2210/pdb5LKE/pdb](http://doi.org/10.2210/pdb5LKE/pdb) |

# Supplementary Figures and Tables

Table S1 Screening of foods used in the reaction between PQQ and glycine*

|  |  | IPQ | Nutritional information (g/100 g food) | | |
| --- | --- | --- | --- | --- | --- |
|  |  | % of control | Protein | Fat | Carbohydrate |
| 1 | Whole milk powder | 219 | 27.1 | 25.5 | 38.9 |
| 2 | Skim milk | 232 | 34.0 | 1.0 | 53.3 |
| 3 | Natural Cheese | 216 | 44.7 | 32.0 | 2.1 |
| 4 | Whey protein | 190 | 93.7 | 0.9 | 2.5 |
| 5 | Butter | 104 | 0.5 | 83.0 | 0.2 |
| 6 | Casein | 133 | 100 |  |  |
| 7 | Lactoglobulin | 98 | 100 | - | - |
| 8 | Lactalbumin | 142 | 100 |  |  |
| 9 | Soy milk | 209 | 3.5 | 3.9 | 2.4 |
| 10 | Tofu (dried) | 208 | 50.0 | 34.7 | 4.5 |
| 11 | Starch | 90 | - | - | 100 |
| 12 | Silica Gel | 101 | - | - | - |
| 13 | Control (buffer) | 100 | - | - | - |

*Reaction conditions: Incubation at 37 °C for 2 h; 1 mL reaction mixture in a 2 mL test tube. The concentrations of the various reaction components were as follows: Food, 1000 mg/L (soymilk was adjusted based on the solid content); PQQ, 50 mg/L; glycine, 250 mg/L; and sodium phosphate buffer (50 mM, pH 7.0). The IPQ yield (HPLC) was 9.7% under the control conditions (no catalyst). The nutritional values listed in the table were obtained using the values listed on the food packages. The skimmed milk composition was obtained from the Japanese Food Composition Table (2020). <https://www.mext.go.jp/a_menu/syokuhinseibun/mext_01110.html>





Figure S1 ^1^H NMR spectra of 10% PQQ in D_2_O under nitrogen and air bubbling conditions.

## Chromatography data of reactions of amino acids with PQQ


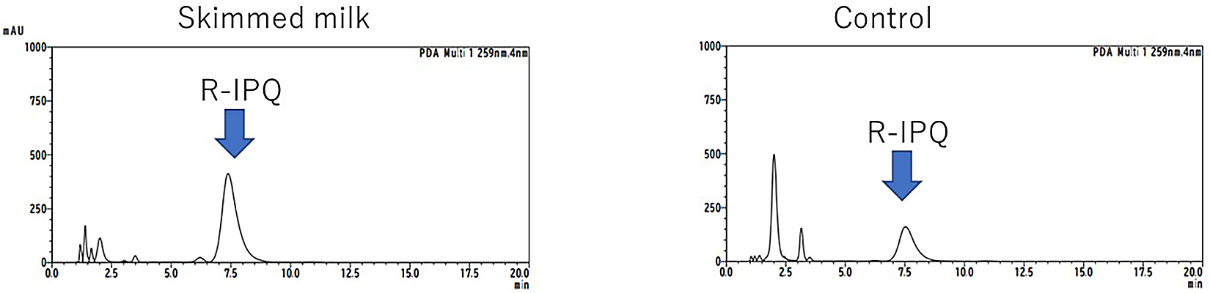


Figure S2-1 Chromatography data for the reaction between PQQ and Leu (HPLC condition 1).


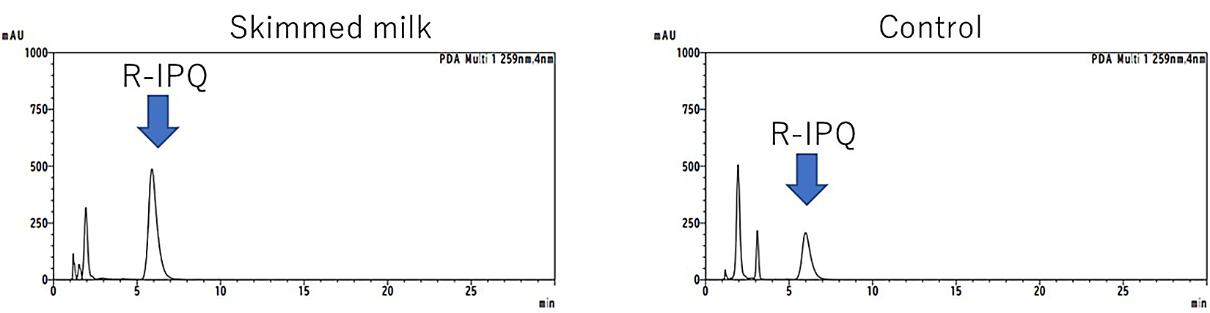


Figure S2-2 Chromatography data for the reaction between PQQ and Ile (HPLC condition 1).


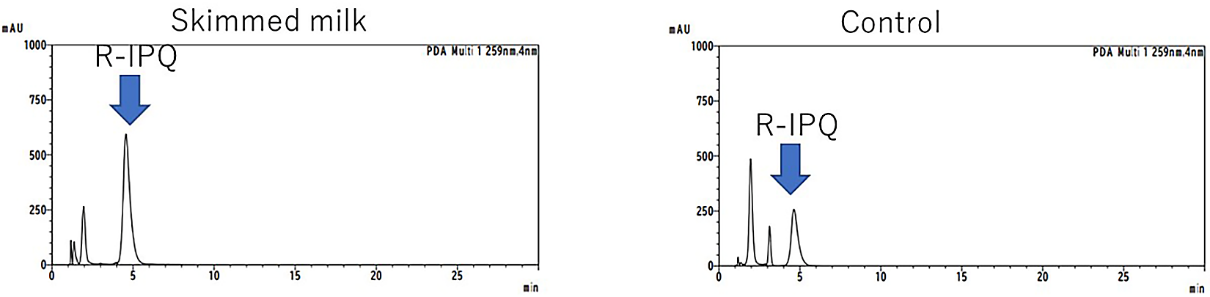


Figure S2-3 Chromatography data for the reaction between PQQ and Val (HPLC condition 1).


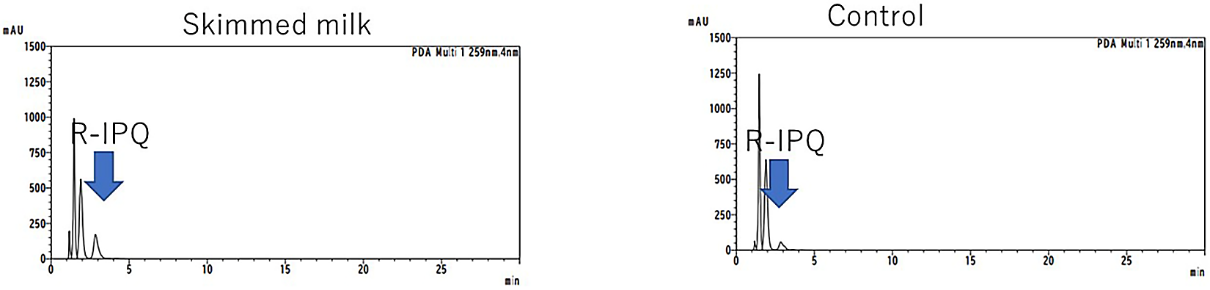


Figure S2-4 Chromatography data for the reaction between PQQ and Asn (HPLC condition 1).


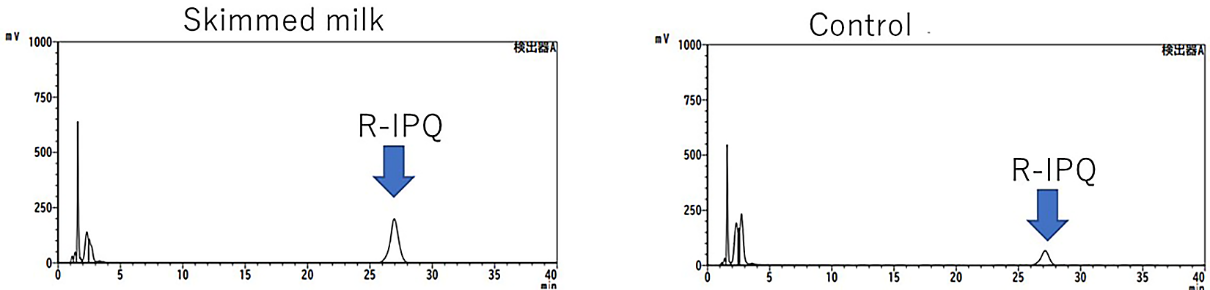


Figure S2-5 Chromatography data for the reaction between PQQ and Arg (HPLC condition 2).

# Supporting Data

Using PQQ and branched-chain amino acids (BCAAs), the effects of various coexisting substances were tested using the coexistence model and the total test process. Derivatization from the normal analytical results was examined for a mixture consisting of PQQ (20 mg) and BCAAs (150 mg; valine 50 mg, leucine 50 mg, and isoleucine 50 mg). Normal analysis was performed using the CB buffer and detected as PQQ. The effects of time immediately after extraction and after overnight incubation at room temperature were examined. The results are shown in Figure S3.





Figure S3 HPLC chromatographs obtained using mixtures of PQQ and BCAAs. a) Derivative analysis of the mixture, indicating 100% PQQ recovery. b) After overnight incubation at room temperature, a recovery of 100% was obtained. c) Normal detection was observed in 74% recovery of PQQ. d) After one night at room temperature, the recovery was further reduced to 67%.

Direct PQQ detection methods have thus far been unable to accurately analyze the presence of BCAAs due to steric hindrance. PQQ have reactivity with BCAAs. Using the current method, only a peak corresponding to PQQ was observed in the direct analysis of PQQ. In contrast, the chromatogram recorded for the mixture of PQQ and BCAA contained a peak corresponding to PQQ and additional peaks at 1, 13, and 16 min, thereby confirming the presence of reactance of BCAA. In the derivative analysis, no changes were observed in the chromatograph, even in the presence of BCAA, thereby indicating that coexisting substances do not influence this analytical method. Indeed, the chromatogram only contained the peak derived from PQQ. Furthermore, the extract was stable, and there was little variation in the result upon changing the analysis start time. Such selective derivatization was possible due to the use of glycine, which exhibits a low degree of steric hindrance (Figure S4).





Figure S4 HPLC analysis of PQQ based on a two-step treatment approach. The extract from the first step was sampled for the second step.

Table S2 Results obtained following analysis of the PQQ-containing (0.1%) chocolate specimen

| Chocolate sample mass (g) | Peak area | PQQ (%) |
| --- | --- | --- |
| 0.4846 | 10307 | 0.100 |
| 0.5124 | 10929 | 0.100 |
| 0.5010 | 10666 | 0.100 |

Table S3 Binding score (kcal/mol) of protein-substrate

|  | 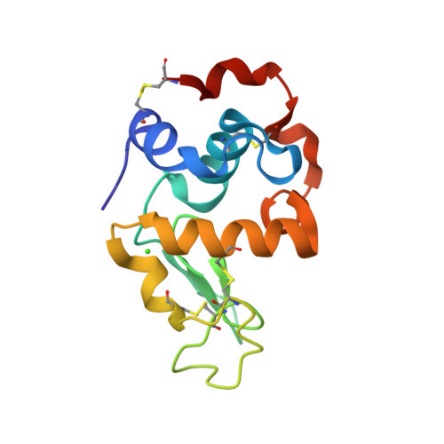  3B0K alpha-lactalbumin | 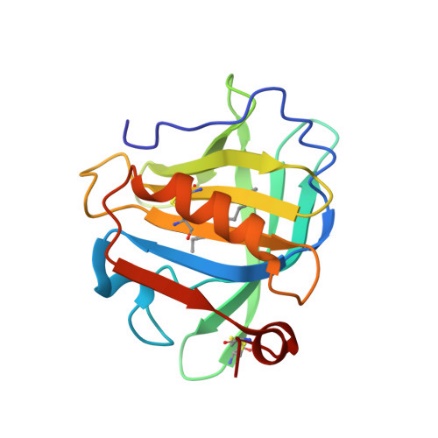  5IO6 Bovine beta-lactoglobulin |
| --- | --- | --- |
| 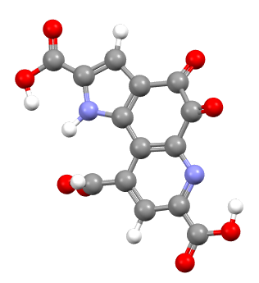  PQQ | -8.0 | -6.1 |
| 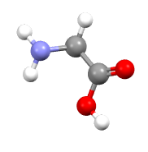Glycine | -3.5 | -3.4 |
| 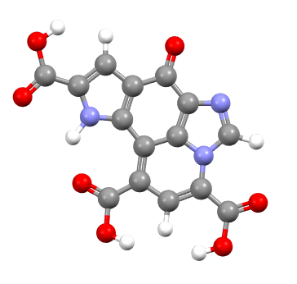  IPQ | -8.0 | -6.6 |

* Docking simulation was performed using Auto dock Vina. The parameters used were the same as in the reference ^3^. Differences in affinity were investigated from docking scores. Lactalbumin was more stable than lactoglobulin in PQQ and IPQ. For glycine, the difference between the two proteins is small. Therefore, adsorption of PQQ easily progresses with lactalbumin. This is the difference in catalytic activity.


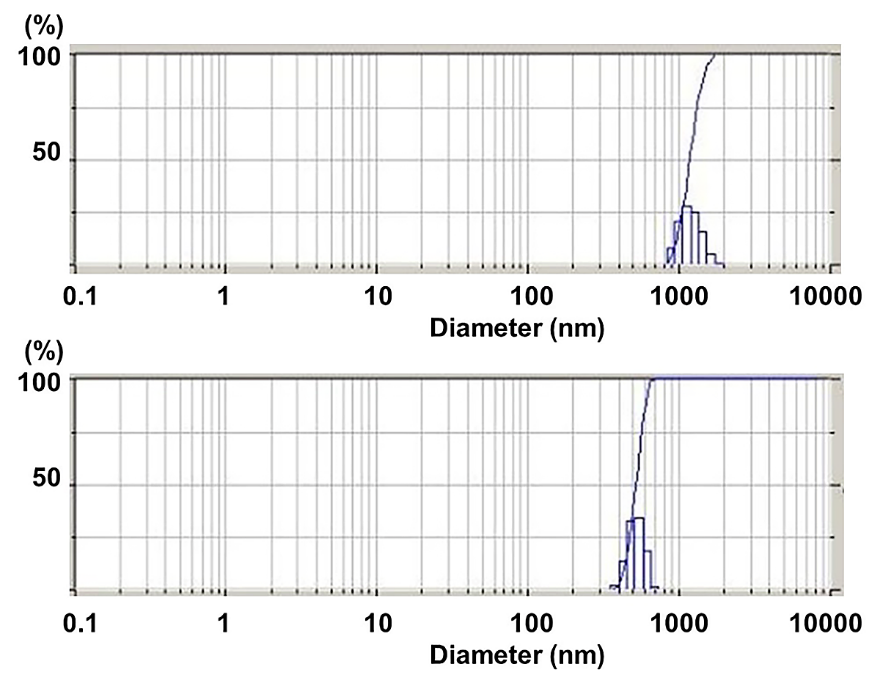


Figure S5 Particle size distributions of the skimmed milk (top) and casein (bottom) specimens, which had average sizes of 1200 and 520 nm, respectively. All measurements were carried out using 100 mg/L of the desired specimen in 50 mM sodium phosphate buffer (HORIBA scientific nanoPartica SZ-100).

# References

1. Urakami, T., Sugamura, K. & Niki, E. Characterization of imidazopyrroloquinoline compounds synthesized from coenzyme PQQ and various amino acids. *BioFactors* **5**, 75–81 (1995).

2. Ikemoto, K., Mori, S. & Mukai, K. Synthesis and crystal structure of pyrroloquinoline quinone (PQQH 2) and pyrroloquinoline quinone (PQQ). *Acta Crystallogr. Sect. B Struct. Sci. Cryst. Eng. Mater.* **73**, 489–497 (2017).

3. Mohamad Ishak, N., Numaguchi T., & Ikemoto K., Antiviral Effects of Pyrroloquinoline Quinone through Redox Catalysis to Prevent Coronavirus Infection, *ACS OMEGA* 47, 44839–44849 (2023).
